# Whole-Genome Sequence of Paenibacillus marchantiae Isolated from the Liverwort Marchantia polymorpha subsp. *ruderalis* Ecotype BoGa

**DOI:** 10.1128/mra.00354-23

**Published:** 2023-06-22

**Authors:** Anja Meierhenrich, Bianca Frommer, Wiebke Halpape, Marvin Hildebrandt, Isabell E. Bleile, Judith Helmig, Sabine Zachgo, Andrea Bräutigam, Bart Verwaaijen

**Affiliations:** a Computational Biology, Faculty of Biology, Bielefeld University, Bielefeld, Germany; b Computational Biology, Centre of Biotechnology (CeBiTec), Bielefeld University, Bielefeld, Germany; c Molecular Biotechnology, Bielefeld University, Bielefeld, Germany; d Botany, School of Biology and Chemistry, Osnabrück University, Osnabrück, Germany; e Department of Genetics, Martin-Luther-University-Halle-Wittenberg, Halle (Saale), Germany; DOE Joint Genome Institute

## Abstract

The bacterium Paenibacillus marchantiae was isolated from male plants of the liverwort Marchantia polymorpha subsp. *ruderalis* ecotype BoGa. Here, we report on the complete genome sequence generated from long Nanopore reads. The genome sequence comprises 6,983,959 bp with a GC content of 46.02% and 6,195 predicted protein-coding genes.

## ANNOUNCEMENT

Species belonging to the genus *Paenibacillus* are rod-shaped and Gram-positive, Gram-variable, or Gram-negative bacteria with an aerobic or facultative anaerobic lifestyle (1, 2). Until today, 285 species are known (3). Back in 1993, the genus was formed from a subgroup of bacilli using 16S rRNA (2). Representatives of *Paenibacillus* have been found in various places, such as in animals (4), rhizospheres (5), leaves (6), and soil (7). Some are known to support plant growth, for example by nitrogen fixation (8), enabling iron uptake by siderophores (9) or through their antimicrobial resistance (10).

Male Marchantia polymorpha subsp. *ruderalis* ecotype BoGa plants (11) were grown in petri dishes on half-strength Gamborg’s medium (Gamborg B5; Duchefa Biochemie B.V., Netherlands) at room temperature under 16-h/8-h day-night conditions. The cetyltrimethylammonium bromide (CTAB) method was applied for DNA extraction using whole plants (12). DNA quality was checked with the Invitrogen Qubit 4 fluorometer (Thermo Fisher Scientific Inc., USA). To prepare DNA for sequencing, the short read eliminator kit (PacBio, USA) and the ligation sequencing genomic DNA (gDNA) kit (SQK-LSK109-XL; Oxford Nanopore Technologies [ONT], Oxford, UK) were used. For sequencing, one R9.4.1 and one R10.0 flow cell were run on a GridION platform, and base calling was performed with the high-accuracy model (MinKNOW v1.4.3; all from ONT). All programs were run with default parameters unless otherwise specified. Genomic reads were checked for contaminations with BLASTN searches (13) against the NCBI nucleotide collection database, and reads matching *Paenibacillus* genome assemblies were filtered (NCBI; Organism “Paenibacillus”; Database “Assembly”; BLAST 2.8.1+; E value < 0.001) (https://www.ncbi.nlm.nih.gov/assembly). *Paenibacillus marchantiae* genome assembly was performed with Canu (v2.2) (14) assuming a genome size of 7.0 Mbp. Racon (v1.4.20) (15), Minimap2 (v2.22-r1101; parameter “–ax map-ont”) (16), and Medaka (v1.4.3; parameter “-m r941_min_high_g360”; ONT) were used for polishing, and Berokka (v0.2.3) (https://github.com/tseemann/berokka) was run for overlap trimming. The assembly resulted in one circular contig (6,983,959 bp; GC content of 46.02%) (Table 1). Benchmarking Universal Single-Copy Orthologs (BUSCO) (v5.4.3; database “bacillales_odb10”) (17) and CheckM (v1.2.2) (18) were applied to check assembly quality resulting in 0.097% contamination and 99.84% assembly completeness. A total of 6,195 protein-coding genes were predicted with Prokka (v1.14.5) (19). Relevant statistics for the assembly and raw reads are listed in Table 1. Metabolic pathways were predicted with the KEGG Automatic Annotation Server (KAAS) (20), GO terms were calculated with eggnog-mapper (v2) (21), and potential secondary metabolite biosynthesis gene clusters were identified with antiSMASH (v6.1.1) (22).

**TABLE 1:** Sequencing and assembly statistics for *Paenibacillus marchantiae*

Parameter	Data
Raw genomic sequencing reads	
No. of reads	404,722
Total length (bp)	3,971,654,859
*N*_50_ (bp)	16,110
Genome sequence	
No. of sequences	1
Length (bp)	6,983,959
GC content (%)	46.02
Genome coverage (×)	236.73
Gene annotation	
Total no. of genes	6,331
No. of protein-coding genes	6,195
No. of rRNAs	33
No. of tRNAs	102
No. of tmRNAs^*a*^	1
BUSCO results (%)	
Complete	98.4
Single copy	98.2
Duplicated	0.2
Fragmented	1.1
Missing	0.5

a tmRNAs, transfer-messenger RNAs.

**FIG 1:**
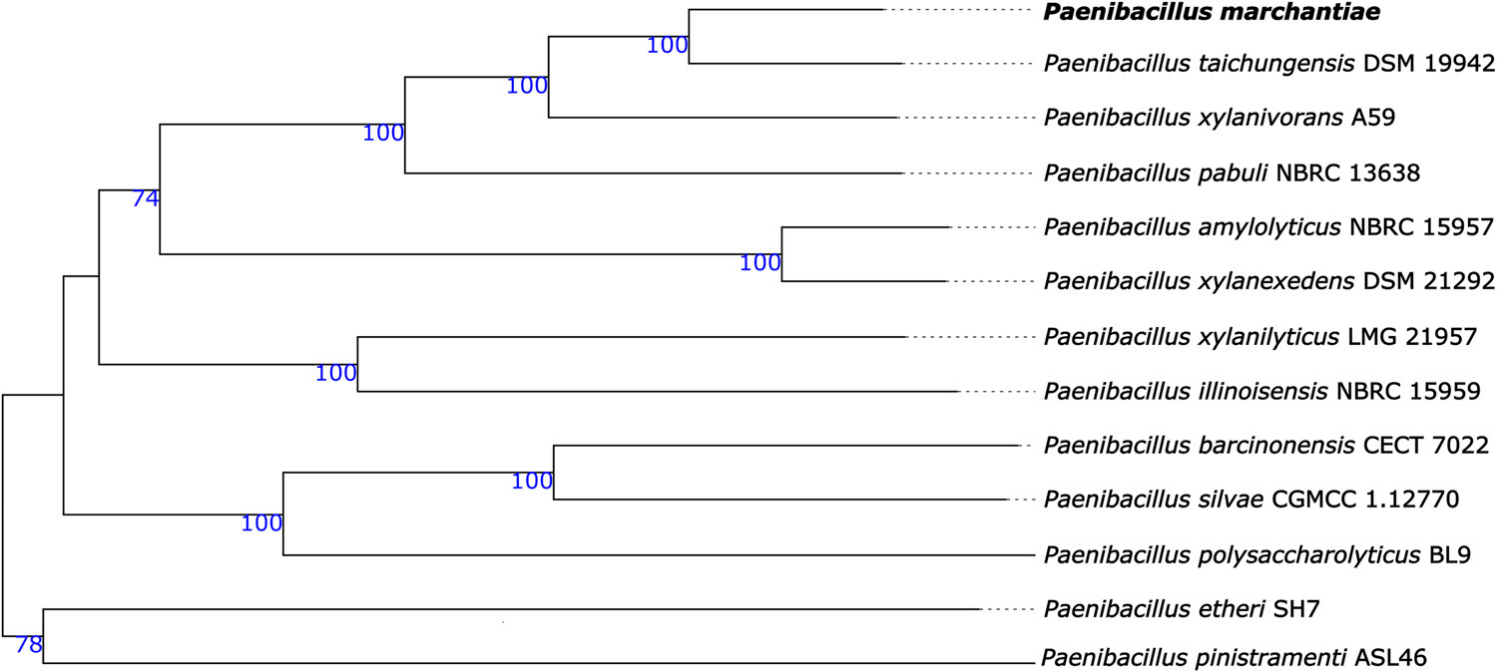
A phylogenetic tree was generated for Paenibacillus marchantiae from the genome sequence with the Type (Strain) Genome Server (TYGS) (22). The lengths of the branches are based on the determination of the GBDP distance formula d5. Above the branches are the GBDP pseudobootstrap support values of >60% from 100 replicates. The mean branch support is 89.9% (22). The phylogenetic tree was aligned at the midpoint. The *Paenibacillus marchantiae* presented here is highlighted in bold type.

Phylogenetic relatedness to other strains was determined using the assembly and Type (Strain) Genome Server (TYGS) (23) (Fig. 1). The closest species were Paenibacillus taichungensis DSM 19942 (GenBank accession number GCA_013359905.1) and Paenibacillus xylanivorans A59 (GenBank accession number GCA_001280595.1), which were both isolated from soil (24, 25).

The KAAS analysis indicates that *Paenibacillus marchantiae* is unable to produce galactose and sucrose but of can take up and degrade them. The gene cluster of paenibactin was identified and comprises all genes for bacillibactin synthesis. These siderophores are responsible for the extracellular conversion of Fe^2+^ and Fe^3+^ and thus enable the provision of iron for plants (9). The metabolic capabilities indicate a potential beneficial partnership between *Paenibacillus marchantiae* and BoGa.

### Data availability.

The genome assembly of *Paenibacillus marchantiae* has been deposited under the NCBI GenBank accession number CP118270.1. The SRA, BioProject, and BioSample accession numbers are SRP421713, PRJNA933122, and SAMN33224160, respectively.
